# Supplementary material for: Role of rosuvastatin and pitavastatin in alleviating diabetic cardiomyopathy in rats: Targeting of RISK, NF-κB/ NLRP3 inflammasome and TLR4/ NF-κB signaling cascades
Source: PLoS One. 2025 Jul 30;20(7):e0325767. doi: 10.1371/journal.pone.0325767 (PMC12310044; doi:10.1371/journal.pone.0325767)
Supplement: S1 File — This file includes additional data and materials that support the findings reported in the main manuscript. It contains supplementary tables, figures, and methodological details referenced in the main text. (DOCX) [file pone.0325767.s001.docx]

**Supplementary File**

1- **Determination of reduced glutathione (GSH)**

The level of reduced glutathione (GSH) was determined in rat cardiac homogenate.

• **Principle**

The method depended on the fact that both protein and non-protein sulfhydral (SH-) groups (mainly GSH) were reacted with Ellman’s reagent [5,5`-dithiobis (2-nitrobenzoic acid)] (DTNB) to form a stable yellow color of 5-mercapto-2-nitrobenzoic acid, which was measured colorimetrically at 412 nm (see the reaction below). In order to determine GSH level in heart tissue, precipitation of protein SH-groups was necessary before the addition of Ellman’s reagent.

• **Reagent**

1. R1. Precipitating solution (trichloroacetic acid)

2. R2. Phosphate solution (0.3M)

3. R3. Ellman’s reagent (DTNB)

• **Procedure**

Protein precipitation in cardiac homogenate: 0.5 ml of the precipitating solution was added to 0. 5 ml homogenate, and to 0.5 ml distilled water to the blank, mixed and allowed to stand for 5 min. After that, followed by centrifugation at 3000 rpm (1000xg) for 15 min. The resultant supernatant was used for the assay of GSH.

Assay: To 0. 5 ml of the resultant supernatant or the blank, 1 ml of phosphate solution was added followed by 0.1 ml of Ellman’s reagent, the absorbance of the resulting yellow color was measured after 5-10 min. at 405 nm using single beam spectrophotometer (Thermospectronic, Helios Gamma, England) against blank.

• **Calculations**

GSH content in cardiac homogenate was expressed as mg/g tissue and was calculated from the following formula:

GSH (nmol/g tissue) = (A S x 22.2) /g tissue used

Where: As= absorbance of the test sample

**2- Determination of lipid peroxidation**

Lipid peroxides formation was determined in rat cardiac homogenate.

• Principle

Lipid peroxidation products were estimated by the determination of the level of thiobarbituric acid reactive substances (TBARS) that were measured as malondialdehyde (MDA). The final was a decomposition product of the process of lipid peroxidation and used as an indicator of this process.

The principle of the assay depended on a colorimetric determination of a pink pigment product, resulting from the reaction of TBARS with thiobarbituric acid in acidic medium, at high temperature95 °C for 30 min and the color was measured at 534 nm wavelength.

• Reagents

1. Chromogen: Thiobarbituric acid, detergent and stabilizer
2. Standard malondialdehyde (MDA) solution

• Procedure

1ml of chromogen was added to blank tube, 0.2 ml sample tissue homogenate or standard solution. Test tubes were covered with glass beads. The mixture was heated for 30 min in the boiling water bath. After cooling, 0.2 ml of sample was added to blank test tube. Mix well, then the absorbance of the pink colored product was measured. Absorbance of sample was measured against blank and the standard against distilled water at 534 nm, using a spectrophotometer (Thermospectronic, Helios Gamma, England).

• Calculations

The concentration of TBARS in cardiac homogenate was expressed as nmol/ml tissue using the following formula:

MDA (nmol/g tissue) = $A Sample 10$

$$\_\_\_\_\_\_\_\_ X \_\_\_\_\_\_\_\_$$

$$A Standard g. tissue used$$

**3- effect size of statistical tests reported as eta-squared**

| **Parameter** | **Effect size (****eta-squared (η²).** |
| --- | --- |
| **Total Cholesterol** | 0.9174 |
| **Triglycerides** | 0.8620 |
| **Glucose** | 0.8719 |
| **RR Interval (s)** | 0.8886 |
| **Heart Rate (BPM)** | 0.9181 |
| **PR Interval (s)** | 0.4448 |
| **QRS Interval (s)** | 0.3874 |
| **QTc (s)** | 0.9548 |
| **R Amplitude (mV)** | 0.6970 |
| **ST Height (mV)** | 0.4609 |
| **NLRP3 (ng/mg protein)** | 0.9515 |
| **Cardiac MDA** | 0.9349 |
| **Cardiac GSH** | 0.7327 |
| **Cardiac pro-fibrotic IL-1 β** | 0.9410 |
| **cardiac Akt** | 0.8627 |
| **p-GSK-3β** | 0.8346 |
| **Cardiac NF-κB** | 0.9833 |
| **TLR-4** | 0.9390 |
| **cardiac troponin** | 0.9591 |

**Supplementary File 1: Detailed Protocol for Masson’s Trichrome Staining**

**Purpose:**

**To detect and visualize collagen deposition and fibrosis in myocardial and aortic tissue sections.**

**Materials:**

1. **Bouin’s solution (for tissue mordanting)**
2. **Weigert’s iron hematoxylin working solution**
3. **Biebrich scarlet-acid fuchsin solution**
4. **Phosphomolybdic-phosphotungstic acid solution**
5. **Aniline blue solution**
6. **1% acetic acid solution**
7. **Ethanol (70%, 95%, absolute)**
8. **Xylene**
9. **Mounting medium**
10. **Distilled water**

**Procedure:**

1. **Deparaffinization and Hydration:**
2. **Place slides in xylene for 2 × 5 minutes.**
3. **Rehydrate through graded alcohols:**
4. **100% ethanol: 2 × 3 minutes**
5. **95% ethanol: 2 minutes**
6. **70% ethanol: 2 minutes**
7. **Rinse in running tap water for 2 minutes.**
8. **Mordanting in Bouin’s Solution:**
9. **Place slides in pre-heated Bouin’s solution at 56°C for 1 hour (or overnight at room temperature).**
10. **Cool to room temperature and rinse in running tap water until the yellow color disappears (5–10 minutes).**
11. **Staining Steps:**
12. **Stain nuclei with Weigert’s iron hematoxylin for 10 minutes.**
13. **Rinse in running tap water for 10 minutes.**
14. **Stain with Biebrich scarlet-acid fuchsin for 10–15 minutes to stain cytoplasm, muscle, and erythrocytes.**
15. **Rinse in distilled water.**
16. **Differentiation:**
17. **Place slides in phosphomolybdic-phosphotungstic acid solution for 10–15 minutes.**
18. **Without rinsing, transfer slides directly to aniline blue solution and stain for 5–10 minutes to visualize collagen fibers.**
19. **Final Differentiation and Dehydration:**
20. **Differentiate in 1% acetic acid for 1–2 minutes.**
21. **Dehydrate through graded alcohols:**
22. **95% ethanol: 2 × 2 minutes**
23. **100% ethanol: 2 × 2 minutes**
24. **Clear in xylene for 2 × 3 minutes.**

**Mounting:**

1. **Mount coverslips with a resinous mounting medium.**
2. **Results Interpretation:**
3. **Collagen fibers: Blue**
4. **Muscle fibers, cytoplasm, erythrocytes: Red**
5. **Nuclei: Black**

**Notes:**

**Timing in staining steps may require optimization depending on tissue thickness and fixation.**

**Proper differentiation is critical to avoid overstaining or understaining of collagen.**
