# Supplementary material for: Role of rosuvastatin and pitavastatin in alleviating diabetic cardiomyopathy in rats: Targeting of RISK, NF-κB/ NLRP3 inflammasome and TLR4/ NF-κB signaling cascades
Source: PLoS One. 2025 Jul 30;20(7):e0325767. doi: 10.1371/journal.pone.0325767 (PMC12310044; doi:10.1371/journal.pone.0325767)
Supplement: Attachment — Submitted filename: Reviewers plos one respone final.docx [file pone.0325767.s003.docx]

**Response to Reviewer Comments**

**Manuscript Title:** Role of Rosuvastatin and Pitavastatin in Alleviating Diabetic Cardiomyopathy in Rats: Targeting of RISK, NF-κB/ NLRP3 Inflammasome and TLR4/ NF-κB Signaling Cascades

**Manuscript ID:** PONE-D-24-48775

We sincerely appreciate the thoughtful and constructive feedback provided by the reviewers. Your insights have greatly enhanced our manuscript, and we are grateful for your time and consideration.

**Editor comment:** 1. Thank you for your response. Before we proceed, please provide additional information regarding the methods of sacrifice.

**Response:** Thank you for drawing our attention to this point. The following sentence was added to the manuscript

The rats were then sacrificed under CO_2_ euthanasia, followed by cervical dislocation, and heart and aorta tissues were dissected.

**Reviewer #1:**

1. **Comment:** 3. Cardiac Contractility: “[ The reported increases and decreases in heart rate, R-R interval, and R-wave amplitude need more context. A comparison between normal, DCM, RVS, and PTS groups could provide additional clarity, particularly regarding baseline values. Stating how RVS and PTS treatments revert or change the observed DCM alterations gives readers a better understanding of the treatment effects.]”

- It would be beneficial to mention the physiological significance of a 16-17% reduction in heart rate, as it presents an opportunity to connect these findings to potential impacts on overall cardiac health.

4. QRS and QTc Interval Analysis:The results indicate significant prolongations in the QRS and QTc intervals associated with DCM. While the shortened intervals following treatment are provided, explaining the clinical relevance of normalizing these indices could reinforce why this research is meaningful in terms of patient care and cardiology.

**Response:**

We appreciate the reviewer's insightful suggestion on the clinical relevance of QRS and QTc normalization. Herein is our response, focusing on the significance of these indices for the management of patients and cardiology in general.

We made these clarifications in the manuscript. The modifications in the manuscript were highlighted in page 19 in the discussion section.

**Revised Explanation:**

Our results unequivocally demonstrated significant lengthening of QRS and QTc intervals with a reduction of the heart rate in the context of DCM, aligning with other studies that identify these parameters as possible indicators of detrimental cardiac remodeling and electrical instability[1–5]. An extended QRS duration indicates ventricular dyssynchrony and conduction abnormalities, often associated with a worse prognosis due to an elevated risk of arrhythmias and abrupt cardiac death. QTc prolongation indicates a delay in cardiac repolarization and increases the risk of potentially fatal arrhythmias, such as torsades de pointes [2,6]. Our investigation recorded reduced QRS and QTc intervals accompanied by a reduction of the heart rate post-treatment, indicating a potential therapeutic impact that may alleviate the arrhythmic load and enhance ventricular synchronization. These data thus corroborate our hypothesis that the normalization of these parameters is associated with improved electrophysiological and structural heart function. This has significant implications for clinical practice, since enhancements in these parameters may lead to decreased morbidity and death in patients with DCM.

2. **Comment: 5. Terminology Consistency: The phrase "counter wise" in line 247 should be corrected to "counterwise" or simply "conversely," as "counter wise" is not standard terminology in scientific writing.**

**Response**: Thank you once again for the opportunity to improve our work. Done, we put the word "conversely," instead "counterwise" in line 247 page 8.

3. **Comment: 8. Role of p-GSK-3β: The results mention reductions in p-GSK-3β levels and an increase in total Akt. Clarifying whether you report total Akt or p-Akt levels (only) would prevent misunderstanding. It would also be prudent to explain the relevance of changing Akt and GSK-3β levels in the context of cardiac health and diabetes.**

**Response**: Thank you for this valuable comment. We confirm that the Akt levels reported in the study refer to total Akt, not phosphorylated Akt (p-Akt). We have clarified this in the revised manuscript to prevent any misunderstanding as follows

Akt, a key regulator in the PI3K/Akt pathway, supports cell survival, growth, and metabolism, while its dysregulation in diabetes impairs cardiomyocyte function and increases apoptosis. GSK-3β, a downstream target of Akt, is active when dephosphorylated, with heightened activity contributing to inflammation, fibrosis, and cardiac remodeling in diabetes. These findings underscore the therapeutic potential of targeting the Akt/GSK-3β axis in diabetic cardiomyopathy [7].

4. **Comment: 9. Gene Expression Analysis: The relationship between NF-κB and TLR-4 should be more explicitly stated. Explain that NF-κB is often a transcription factor that could enhance the expression of pro-inflammatory genes, including TLR-4. Providing a clearer causal pathway of how inflammation contributes to DCM could strengthen the findings.**

**Response**: Thank you for this insightful comment. In the revised manuscript, we have explicitly detailed the relationship between NF-κB and TLR-4 as follows:

“NF-κB, as a transcription factor, enhances the expression of pro-inflammatory genes like TLR-4. Hyperglycemia-induced stress activates NF-κB, leading to its translocation to the nucleus, where it upregulates inflammatory genes, creating a feedback loop that sustains chronic inflammation. This process contributes to the pathogenesis of diabetic cardiomyopathy (DCM) by mediating cardiac inflammation, fibrosis, and structural remodeling, ultimately impairing cardiac function. Understanding this causal pathway highlights the importance of targeting the TLR-4/NF-κB axis for potential therapeutic intervention in DCM [8].”

**- Discussing the functional implications of a 6-fold increase in NF-κB alongside the 4.5-fold increase in TLR-4 would enhance the understanding of how inflammation exacerbates diabetic cardiomyopathy.**

**Response**: Your insights have greatly enhanced our manuscript, and we are grateful for your time and consideration. It has been added as follows:

A 6-fold increase in NF-κB, coupled with a 4.5-fold increase in TLR-4, as represented in the current study, indicates a significant upregulation of the inflammatory response in DCM. TLR-4 activation triggers a cascade of downstream proteins, including NF-κB, which enhances the expression of pro-inflammatory genes, sustaining chronic inflammation in the myocardium. This pathway contributes to the pathogenesis of DCM by promoting cardiac inflammation, fibrosis, and structural remodeling, ultimately impairing cardiac function. Additionally, activation of the NLRP3 inflammasome amplifies the inflammatory response through cytokines like IL-1β and IL-18, worsening myocardial injury. Targeting the TLR-4/NF-κB axis and NLRP3 inflammasome presents a potential therapeutic strategy to mitigate inflammation and prevent DCM progression in diabetic patients.

5. **Comment: 10. Troponin Levels:**

**-The text states, "diabetic rats presented a notable boost in levels of cardiac troponin compared to normal control by 91%." It would be helpful to provide the absolute values of cardiac troponin levels for both the normal control and DCM groups for more context and clearer comparison.**

**Response**:

Done, we edit this sentence as follows “Likewise, diabetic rats exhibited a notable increase in cardiac troponin levels, rising from 20.24 to 38.66 mg/tissue protein compared to the normal control group.”

**- The phrase "RSV and PTS treatment presented markedly improvement" should be reworded to "RSV and PTS treatment resulted in marked improvements" for grammatical accuracy and clarity.**

**Response**:

Done, we added the revised sentence "RSV and PTS treatment resulted in marked improvements" in line 328 on page 12

6. **Comment: 11. Histopathological Examinations:**

**- The description beginning with "In myocardial sections of the normal control group" could be structured for better flow. For example, starting with, "Histopathological examinations revealed normal myocardial architecture in the control group, as illustrated in figure 9..."**

**Response**: Done, thank you. The modified sentence was added in lines 34 and 341 on page 13.

**- The phrase "showed The myocardial accumulation of" should be corrected to "showed myocardial accumulation of" to remove the grammatical error.**

**Response**: Done in line 343 page 13.

**- Clarifying the term "fat deposits" by consistently referring to how it relates to the DCM pathology may enhance understanding. For instance, defining the significance of these fat deposits in the context of diabetic cardiomyopathy could provide clarity.**

**Response**: Thank you for the suggestion. To improve clarity, the term "fat deposits" has been more explicitly linked to the pathology of diabetic cardiomyopathy (DCM) as follows:

“Moreover, the F/Fr/STZ-induced DCM model exhibited marked myocardial accumulation of infiltrating inflammatory cells, along with collagen deposition in cardiac myocytes, indicative of evident fibrosis between the cardiac muscle fibers. This was accompanied by fat deposits within the myocardium, which are commonly associated with myocardial lipid infiltration, a characteristic feature of metabolic disorders like diabetes. The buildup of fat within the myocardium disrupts normal cardiac function, contributing to inflammation, fibrosis, and impaired contractility—key characteristics of DCM [9].

**12. Treatment Effects on Myocardial Architecture:**

**- It is noteworthy that the RVS-treated group showed “normal myocardial architecture.” However, it could be beneficial to describe what aspects of the architecture (e.g., fiber arrangement, size of the cells) were restored to normal or near-normal appearance.**

**Response**: Thank you for the suggestion. To improve clarity, the description of "normal myocardial architecture" can be expanded to specify the aspects of the cardiac structure that were restored in the RVS-treated group. Here's an updated version:

“It has shown a normal myocardial architecture, i.e. normal arrangement of myocardial fibers, organized in a parallel and uniform pattern, and the size of cardiac myocytes was normalized without signs of hypertrophy or atrophy.”

**- For comparison, specifying how the architecture in the PTS-treated group differs from both the normal and DCM groups would help elucidate the treatment effects (e.g., "PTS-treated rats also demonstrated a restoration to normal architecture, although some subtle differences were noted compared to the normal group.").**

**Response**: To enhance clarity and specify the differences in myocardial architecture between the PTS-treated group, the normal group, and the DCM group, the following revision can be made:

“The treated groups showed a myocardial fiber arrangement similar to the healthy control group, though with slight irregularities in fiber alignment. The cardiac myocyte size was slightly larger than the normal group but still smaller than in the DCM group, indicating restoration of myocardial structure.”

**13. Masson’s Trichrome Staining:**

**- When discussing fibrotic changes, it would be beneficial to briefly explain the significance of Masson’s Trichrome staining for readers who might not be familiar with the technique. Highlighting why fibrosis is important for understanding DCM could enhance the importance of these findings.**

**Response**: Thank you for the suggestion. Here's an improved version with an explanation of Masson’s Trichrome staining and its significance in understanding DCM:

“Masson's Trichrome staining was used to assess fibrosis collagen deposit in the myocardium. This staining technique specifically highlights collagen fibers, which are key markers of fibrosis. In this study, Masson’s Trichrome staining revealed increased collagen deposition in the DCM group, indicating the presence of myocardial fibrosis. Fibrosis is an important pathological feature of DCM, as the excessive deposition of collagen disrupts the normal architecture and function of the heart. The accumulation of fibrotic tissue can impair myocardial contractility, promote cardiac remodeling, and ultimately lead to heart failure. Therefore, the identification of fibrosis through Masson’s Trichrome staining underscores the severity of DCM and the potential of treatments, like PTS, to mitigate these pathological changes.”

**14. Aortic Tissue Changes:**

**- The statement “hypertrophied aortic wall of the DCM control group” could be elaborated. Perhaps include metrics like thickness measurements if available.**

**- The note about RVS preventing "approximately 50% of aortic wall thickening" could be further clarified with the actual measurements of wall thickening for a more objective understanding.**

**Response**: Done

As follows:

“However, the aortas of diabetic rats showed hypertrophied aortic walls with increased thickness (mean = 123.97 µm) due to medial collagen accumulation, likely resulting from increased dietary fat intake and STZ administration (Fig. 11C, D). Diabetic rats treated with RVS demonstrated a reduction in media thickness compared to the untreated diabetic group (mean = 98.59 µm) (Fig. 11E, F). Notably, treatment with the PTS restored the aortic appearance to near-normal, with a media thickness close to that of the control group (mean = 73.38 µm) (Fig. 11G, H).

Additionally, cardiomyocyte diameter was measured at ×400 magnification as illustrated in figure 11I, showing differences between groups. The mean diameter in the normal control group was 13.42 µm, while it increased to 21.84 µm in the untreated diabetic group. Treatment with RVS reduced the diameter to 16.49 µm, and PTS further reduced it to 15.45 µm.”

**15. Caspase-1 Immunohistochemistry:**

**- The phrase "the overall fibrosis was identified" can be more decisively stated. Instead, consider, "The extent of fibrosis was quantified..."**

**Response**: Thank you for the suggestion. Here's the revised response to the reviewer comment:

“The extent of fibrosis was quantified in each of the examined hearts, and the average amount of fibrosis is summarized in Table 2. In the Normal Control group, no fibrosis was observed (0). The DCM group exhibited significant fibrosis (+++), while treatment with PTS (DC+PGZ) reduced fibrosis to a mild degree (+), and RVS treatment (DC+RVS) showed moderate fibrosis (++) compared to the DCM group. These findings highlight the varying degrees of fibrosis and underscore the potential effectiveness of the treatments in mitigating fibrotic changes in DCM.”

**- Detailing the significance of high caspase-1 expression in DCM pathology may clarify why it is important in this context. Discussing caspase-1's role in inflammation or apoptosis could elevate the findings.**

**Response**: To clarify the significance of high caspase-1 expression in diabetic cardiomyopathy (DCM) pathology, the following explanation can be added:

“High caspase-1 expression is crucial in the pathogenesis of DCM due to its role in inflammasome activation and the subsequent inflammatory response.

**Grammatical and Stylistic Review**

**1. Structure and Flow:**

**- The section lacks smooth transitions between different sub-sections (3.1 and 3.2). It would improve readability to introduce each subsection with a brief rationale or connective sentence linking metabolism and cardiac function.**

**Response**: Done on page 8

**- Enhance the transitions between different sections (e.g., from oxidative stress to inflammatory signaling) to improve the flow. For example, include phrases like “Furthermore, we also investigated...” can make the narrative smoother.**

**Response**: Done on page 10

 I added clearer connections between related topics, such as oxidative stress and inflammatory signaling.

**2. Punctuation and Spacing:**

**- Consistent spacing around units (e.g., “30 %” should be “30%”) should be maintained throughout to enhance readability.**

**- Improving punctuation surrounding phrases like "and TG levels, by approximately 2.7-, 2.0-, and 2.1-fold," is recommended. A clean, consistent format without redundant punctuation would help in clarit**

**- The expression "when related to" is somewhat awkward. Alternatives such as "compared to" or "relative to" can make the text sound more polished.**

**- Keep consistency in formatting. For example, “cardiac content of IL-1β, by about 3.2- and 2.9-fold” should uniformly present the fold changes—either with “about” or without throughout the text.**

**Response**: Done

**3. Consistency of Presentation:**

**- Ensure consistency in how percentages are presented. For instance, use either "X%" throughout or spell out terms as "X percent." This uniformity avoids confusion for the reader.**

**Response**: Done

**4. Redundant or Unclear Phrasing:**

**- The phrase "a decline in the elevated TC and TG significantly by 61%, 58%, and 57%, 45%," is awkwardly structured. It could be rephrased for clarity: “treatment with RVS and PTS led to significant declines in elevated TC and TG levels, by 61% and 58%, and by 57% and 45% respectively.”**

**- Avoid using terms like "showed" multiple times in close proximity. Instead of starting each finding with "showed," consider varying the sentence beginnings, such as “The results indicated...” or “Data revealed...”.**

**- Use consistent language to describe findings. For example, terms like "showed," "demonstrated," and "indicated" can be alternated appropriately to enhance readability.**

**Response**: Done

**- Modify "marked positivity in DCM group with improvement in treated groups" to "demonstrated marked positivity in the DCM group, with observed reductions in the treated groups."**

**Response**: Done

**- The phrase “provoked the inflammatory cascades that proved by a marked elevation” in line 296 is somewhat confusing. A clearer phrasing might be, "The F/Fr/STZ model induced significant inflammatory cascades, evidenced by a marked elevation in Akt levels and a reduction in p-GSK-3β."**

**Response**: Done

**- “Signifying that the inflammatory cascades were initiated and exacerbate the cardiomyopathy diabetic complication” needs rephrasing for better clarity. For example, “indicating the initiation and exacerbation of diabetic cardiomyopathy due to inflammatory cascades.”**

**Response**: Done

**- Simplify and streamline sentences where possible. For instance, “This was correlated with the accumulation of fat deposits within the myocardium” can be shortened to “This correlated with fat deposits in the myocardium.”**

**Response**: Done

**5. Consolidation of Findings:**

**- The overall descriptions could benefit from a concluding sentence summarizing the key findings. This reinforces the importance of the data presented.**

**Response:** Thank you for your feedback. The sentence you requested is already included in the conclusion:

"This study demonstrates the prominent cardioprotective potential of both RSV and PTS in the context of DCM in rats. These statins effectively improved cardiac function, reduced oxidative stress, and mitigated inflammatory pathways, including NLRP3 inflammasome and NF-κB/TLR‑4 signaling, as well as the RISK signaling pathway. Molecular signaling imbalances in DCM were also addressed by the statin treatments."

**V. Discussion:**

**Scientific Review**

**1. Mechanistic Clarity and Relevance:**

**- Lack of Novelty: The discussion reiterates well-established mechanisms of DCM (e.g., oxidative stress, TLR4/NF-κB pathway, AGEs) without providing new insights. The authors should emphasize how their findings contribute to the existing body of knowledge or highlight mechanisms specific to their study that have not been previously addressed.**

**Response:** Thank you for your valuable feedback. We acknowledge that certain mechanisms underlying DCM, such as oxidative stress and the TLR4/NF-κB pathway, are well-established in the existing literature. However, our study provides new insights by examining the specific involvement of these pathways in the context of the F/Fr/STZ-induced DCM rat model and evaluating the therapeutic effects of RSV and PTS. In particular, we highlight the significant upregulation of NF-κB and TLR4, which plays a key role in amplifying the inflammatory responses in DCM. Additionally, we introduce the novel aspect of the interplay between AGE-induced ROS production and the activation of inflammatory pathways, a connection that has not been fully explored in prior studies. Furthermore, we elaborate on the maladaptive effects of the Akt/GSK3β pathway in DCM, contributing new mechanistic insights into the disease process. Our findings support the therapeutic potential of statins like RSV and PTS in targeting these pathways, providing important implications for clinical applications in managing diabetic cardiomyopathy.

**- Need for Citations: Assertions about the role of ROS and AGE s in DCM need better citations. Statements regarding oxidative stress and its role in cardiac dysfunction and hypertrophy should reference more recent literature or landmark studies to corroborate claims (e.g., publications from the last 3-5 years).**

**Response:** we have added the following references

Xu, N., Liu, S., Zhang, Y., Chen, Y., Zuo, Y., Tan, X., … Feng, J. (2023). Oxidative stress signaling in the pathogenesis of diabetic cardiomyopathy and the potential therapeutic role of antioxidant naringenin. Redox Report, 28(1). <https://doi.org/10.1080/13510002.2023.2246720>

Molecular mechanisms of metabolic dysregulation in diabetic cardiomyopathy <https://doi.org/10.3389/fcvm.2024.1375400>

**- Causative Link: The connection between AGEs and the activation of inflammatory pathways should be drawn more clearly. It is not sufficient to state correlations; the authors must discuss potential causal relationships supported by the literature.**

**Response:** We appreciate the reviewer’s insightful observation regarding the need to clarify the causative relationship between AGEs and the activation of inflammatory pathways. In the revised manuscript, we have expanded the discussion to emphasize the mechanistic basis for this connection, supported by recent literature.

Chronic hyperglycemia enhances the production of advanced glycation end products (AGEs), which leads to oxidative overload, increased ROS generation, and the amplification of oxidative stress. AGEs interact with the receptor for advanced glycation end products (RAGE) on the cell membrane, triggering downstream signaling cascades such as MAPK, p38, SAPK/JNK, ERK1/2, and JAK/STAT. These pathways activate transcription factors, particularly NF-κB, which subsequently induce the expression of pro-inflammatory cytokines (e.g., TNF-α, IL-1β, IL-6) and apoptosis-related genes. This sequence of events promotes inflammation, cell death, and tissue damage, all of which are hallmarks of DCM pathology [10].

**- Lines 474-476: The section discussing the **RISK pathway** and **Akt/GSK3β** signaling appears convoluted. The authors should provide a more explicit connection between hyperglycemia, the upregulation of Akt, and the resulting physiological outcomes. This could be enhanced by defining the RISK pathway early in the discussion to ground the reader in its relevance and components.**

**- Lines 474-476: A more structured presentation of the **beneficial and detrimental effects of Akt activation** would greatly aid comprehension. A table or bullet points outlining these effects could help convey the dual nature of Akt signaling more effectively.**

**Response:** This section of the discussion has been revised for greater precision and clarity, with additional elaboration as follows:

**“**The signaling cascade was further validated by examining the intermediary protein RISK pathway involving Akt/GSK-3β, which is closely associated with the adverse effects of hyperglycemia. Additionally, our study elucidates the dual role of the Akt/GSK3β pathway in DCM. While Akt activation is typically protective, chronic activation in the context of hyperglycemia exacerbates cardiac fibrosis and hypertrophy, contributing to long-term cardiac dysfunction. This nuanced understanding of the Akt/GSK3β pathway in DCM progression offers new therapeutic targets for managing the condition. Akt, a key regulator in the PI3K/Akt pathway, promotes cell survival, growth, and metabolism. However, in diabetes, particularly in DCM, chronic hyperglycemia and AGEs upregulate Akt activity, leading to diverse cardiac outcomes [53]. While Akt activation can protect cardiac cells from apoptosis, promote cell survival, and support adaptive responses under stress, persistent and excessive activation can lead to maladaptive effects, including cardiac hypertrophy, fibrosis, and contractile dysfunction, ultimately contributing to cardiac dysfunction and heart failure. GSK-3β, a downstream target of Akt, is active in its dephosphorylated form, and its heightened activity exacerbates inflammation, fibrosis, and cardiac remodeling in diabetes. These findings underscore the dual role of Akt activation and highlight the therapeutic potential of targeting the Akt/GSK-3β axis in managing diabetic cardiomyopathy [53,54]”.The detrimental effects caused by persistent and excessive Akt activation can lead to maladaptive responses, contributing to cardiac hypertrophy, fibrosis, and contractile dysfunction. These changes can eventually cause cardiac dysfunction and heart failure [13,14].

**3. TLR4/NF-κB Cascade:**

**Response:** This section of the discussion has been revised for greater precision and clarity, with additional elaboration as follows:

“NF-κB, as a transcription factor, enhances the expression of pro-inflammatory genes like TLR-4. Hyperglycemia-induced stress activates NF-κB, leading to its translocation to the nucleus, where it upregulates inflammatory genes, creating a feedback loop that sustains chronic inflammation. This process contributes to the pathogenesis of diabetic cardiomyopathy (DCM) by mediating cardiac inflammation, fibrosis, and structural remodeling, ultimately impairing cardiac function. Understanding this causal pathway highlights the importance of targeting the TLR-4/NF-κB axis for potential therapeutic intervention in DCM [8]. A 6-fold increase in NF-κB, coupled with a 4.5-fold increase in TLR-4, as represented in the current study, indicates a significant upregulation of the inflammatory response in DCM. TLR-4 activation triggers a cascade of downstream proteins, including NF-κB, which enhances the expression of pro-inflammatory genes, sustaining chronic inflammation in the myocardium. This pathway contributes to the pathogenesis of DCM by promoting cardiac inflammation, fibrosis, and structural remodeling, ultimately impairing cardiac function. Additionally, activation of the NLRP3 inflammasome amplifies the inflammatory response through cytokines, worsening myocardial injury. Targeting the TLR-4/NF-κB axis and NLRP3 inflammasome presents a potential therapeutic strategy to mitigate inflammation and prevent DCM progression in diabetic patients.”

**- The discussion on TLR4/NF-κB activation appears disjointed. Transitioning smoothly between the introduction of TLR4 and its downstream effects could improve readability. For example, ensuring that the links between activation and subsequent inflammatory processes are cohesive would enhance understanding.**

**- While TLR4-related pathways are acknowledged, details regarding the molecular mechanisms (e.g., specific cytokines involved in the cascade, feedback loops) need elaboration to demonstrate depth of understanding.**

**Response:** This section of the discussion has been revised for greater precision and clarity, with additional elaboration as follows:

“The signaling cascade was further validated by examining the intermediary protein RISK pathway involving Akt/GSK-3β, which is closely associated with the adverse effects of hyperglycemia. Additionally, our study elucidates the dual role of the Akt/GSK3β pathway in DCM. While Akt activation is typically protective, chronic activation in the context of hyperglycemia exacerbates cardiac fibrosis and hypertrophy, contributing to long-term cardiac dysfunction. This nuanced understanding of the Akt/GSK3β pathway in DCM progression offers new therapeutic targets for managing the condition. Akt, a key regulator in the PI3K/Akt pathway, promotes cell survival, growth, and metabolism. However, in diabetes, particularly in DCM, chronic hyperglycemia and AGEs upregulate Akt activity, leading to diverse cardiac outcomes [15]. While Akt activation can protect cardiac cells from apoptosis, promote cell survival, and support adaptive responses under stress, persistent and excessive activation can lead to maladaptive effects, including cardiac hypertrophy, fibrosis, and contractile dysfunction, ultimately contributing to cardiac dysfunction and heart failure. GSK-3β, a downstream target of Akt, is active in its dephosphorylated form, and its heightened activity exacerbates inflammation, fibrosis, and cardiac remodeling in diabetes. These findings underscore the dual role of Akt activation and highlight the therapeutic potential of targeting the Akt/GSK-3β axis in managing diabetic cardiomyopathy [11,12]. The detrimental effects caused by persistent and excessive Akt activation can lead to maladaptive responses, contributing to cardiac hypertrophy, fibrosis, and contractile dysfunction. These changes can eventually cause cardiac dysfunction and heart failure [13,14].”

**4. Inflammasome Discussion:**

**- The paragraph discussing NLRP3 needs strengthening. It would be beneficial to provide context for how NLRP3 relates specifically to the pathophysiology of DCM beyond its general inflammatory role. Are there specific signaling pathways or molecular alterations observed in the current study that implicate NLRP3 more directly in DCM?**

**Response:** Thank you for your insightful comment. We agree that strengthening the discussion around NLRP3 is important to provide a more specific context regarding its role in DCM pathophysiology.

“The overactivation of NLRP3 was accompanied by elevated levels of downstream cytokines, which exacerbate myocardial injury by amplifying local inflammatory responses. Additionally, the activation of NLRP3 was shown to synergize with the TLR4/NF-κB pathway, as evidenced by the observed upregulation of TLR4 expression and NF-κB nuclear translocation. This cross-talk between NLRP3 and NF-κB further perpetuates a pro-inflammatory environment, promoting cardiac fibrosis and structural remodeling [16]”

**- The term “punch of pro-inflammatory” is awkward and unclear. A more formal term such as "a surge of pro-inflammatory cytokines" should be used instead.**

**Response: Done**

**5. Potential Treatment Effects:**

**- When discussing the effects of RVS and PTS, quantitative data or specific findings would enhance credibility. For instance, specifying the extent of decrease in cardiac markers or inflammation would provide a clearer picture of treatment efficacy. Broad statements like "alleviated the cardiac changes" require quantitative backing to assess the clinical relevance of the findings.**

**Response: Done**

**- The mention of previous studies (e.g., Liu et al.) must be tied back to the current findings with more specificity. What mechanisms were examined in that study, and how do they align with the outcomes observed here?**

**Response: Done**

**We wrote in manuscript “**A recent study conducted by Liu *et al.* suggests that RVS administration in post-conditioning can reduce the severity of cardiac damage caused by ischemia and reperfusion in isolated hearts [17].

Rosuvastatin prevents myocardial ischemia–reperfusion injury by inducing phosphorylation of PI3K–Akt and GSK-3β, concomitant with a higher Ca2+ load required to open the mPTP preventing oxidative stress and subsequent inhibition of mPTP opening. Moreover, Rosuvastatin postconditioning also significantly increased superoxide dismutase activity and reduced malondialdehyde and radical oxygen species level. “

**7. Contradictory Effects of Statins:**

**- Lines 492-498: The discussion of statins' effects, particularly their dual role in inflammation, is somewhat contradictory and lacks nuance. The authors should clarify how statins can both reduce and potentially augment IL-1β expression. Providing specific studies that detail these opposing actions with mechanisms would strengthen this section.**

**Response: Done**

To address the contradictory nature of statins' effects on IL-1β expression, the authors can clarify the dual role by distinguishing the contexts under which these opposing effects occur. The following revision provides a more nuanced explanation, incorporating specific studies and mechanisms:

"Statin therapy has been observed to have a dual impact on inflammation-related factors, particularly IL-1β. On one hand, statins can reduce the expression of NLRP3 and downstream mediators like IL-1β, which are significant contributors to inflammation in atherosclerosis. For instance, one study demonstrated that statins decreased NLRP3 gene expression in peripheral blood mononuclear cells from patients with cardiovascular disease, suggesting their anti-inflammatory potential under certain conditions [18]. This reduction in IL-1β expression is thought to be related to statins' ability to inhibit the activation of pro-inflammatory pathways, including NF-κB and the inflammasome, which are involved in the production of IL-1β during inflammatory responses.

On the other hand, several studies have indicated that statins can augment the expression of IL-1β when lipopolysaccharides (LPS) are present, particularly in the absence of NLRP3 inflammasome activation. For example, a study by Sheridan et al., 2022 demonstrated that statins enhanced IL-1β expression in LPS-stimulated macrophages [19], suggesting that the inflammatory response to pathogens could be exacerbated under certain conditions [20]. This increase in IL-1β expression has been attributed to statins' potential to activate alternative inflammatory pathways, such as the upregulation of the NF-κB pathway independent of NLRP3 inflammasome activation, or the modulation of TLR4 signaling, both of which could increase IL-1β production in the presence of an immune stimulus.Thus, the effects of statins on IL-1β expression appear to be context-dependent, varying according to the specific inflammatory environment, the presence of pathogens, and the balance between inflammasome activation and NF-κB signaling. These opposing actions highlight the complexity of statins' inflammatory modulation, underscoring the need for further studies to elucidate the precise mechanisms that govern these effects and their implications for cardiovascular therapy."

This revised section not only clarifies the contradictory nature of statins' effects but also provides studies and mechanisms for each opposing action, giving readers a better understanding of the context in which these effects may occur.

**- Line 494: There is a mention of an increase in IL-1β in response to lipopolysaccharide stimulation when NLRP3 is inactive. This should be explained further: how do statins mediate this effect, and what are the practical implications regarding their use in inflammatory conditions in cardiovascular patients?**

**Response: Done**

**As previously mentioned**

**8. Lack of Specificity in Claims:**

**- Line 482: The phrase "the exact mechanisms are still under investigation" is vague. If the authors are aware of ongoing research or have preliminary findings, they should specify these to enhance depth. A sentence summarizing the current understanding while acknowledging the research gaps would enrich this section.**

**Response:** You're absolutely right that the phrase "the exact mechanisms are still under investigation" could be more specific to add depth and clarity. A more informative approach would be to provide a summary of the current understanding while acknowledging the gaps in research. Here’s an enhanced version: “Although the mechanisms through which statins influence the RISK pathway and modulate oxidative stress are still being explored, several key insights have emerged from ongoing research.”

**- Lines 499-501: The statement regarding **thioredoxin-1 and HO-1** as antioxidant enzymes requires more elaboration on how statins are proposed to modulate these enzymes in the context of DCM and the broader implications of these effects in cardiac physiology.**

**Response:** The following expanded explanation could be added to better clarify the role of **thioredoxin-1** and **heme oxygenase-1 (HO-1)** in the context of statin therapy as follows:

“Statins, including rosuvastatin (RVS), modulate key antioxidant enzymes such as thioredoxin-1 and heme oxygenase-1 (HO-1), which play crucial roles in protecting the heart from oxidative stress in DCM. Statins enhance thioredoxin-1 activity by promoting its S-nitrosylation, reducing intracellular ROS and oxidative damage. They also upregulate HO-1 expression through the PI3K/Akt pathway, further alleviating oxidative stress and preventing myocardial fibrosis, hypertrophy, and dysfunction. These pleiotropic effects of statins, beyond their lipid-lowering action, have important therapeutic implications in protecting cardiac tissues from oxidative injury in DCM.”

**9. Strength of Evidence:**

**- Lines 483-484: Several claims, such as “statins may influence the activity of certain proteins or enzymes involved in the RISK cascade,” lack empirical support within this text. It would be beneficial to include citations for these assertions or mention representative studies to bolster credibility.**

**Response:**

Thank you for your valuable feedback. We acknowledge the reviewer’s concern regarding the lack of empirical support for the statement that “Although the mechanisms through which statins influence the RISK pathway and modulate oxidative stress are still being explored, several key insights have emerged from ongoing research, as illustrated in figure 14. Statins may enhance the cell’s ability to withstand reperfusion-associated stress, thereby minimizing damage to heart tissue. Specifically, statins are known to influence proteins and enzymes involved in the RISK cascade, with studies demonstrating that statins modulate key signaling molecules such as PI3K, Akt, and GSK-3β, which are integral to myocardial protection during ischemia-reperfusion. For example, research by Bland et al. (2022) has shown that statins enhance Akt phosphorylation, activating the RISK pathway and protecting the heart from oxidative stress and reperfusion injury [21]”

**- Lines 504-506: When citing previous studies regarding the effect of statins on oxidative stress and arrhythmias, the authors should provide more context about the study designs and sample sizes to give the claims weight.**

**Grammatical and Stylistic Review**

**1. Structural Organization:**

**- The overall flow is disjointed. Each paragraph should logically follow the previous one, maintaining a coherent narrative thread throughout the discussion. Consider restructuring for better logical sequencing. For example, begin with the significance of DCM and then gradually introduce your findings, linking them back to existing literature throughout.**

**Response: Done**

**- Line 475: Some sentences are overly long and complex, making them challenging to follow. For example, the sentence starting with "This elevation in Akt signaling can be triggered by various factors..." can be broken down into shorter sentences to enhance readability.**

**Response: Done**

**- Lines 474-475: Consider restructuring sentences to prioritize subject-verb-object construction, which is generally clearer. For example: “Chronic hyperglycemia and AGEs trigger the elevation in Akt signaling, leading to various cardiac outcomes.”**

**Response: Done**

**- Line 493: Phrases like “which were directly related to the adverse effects of hyperglycemia” could be simplified. A clearer rephrasing might be, “which are associated with the adverse effects of hyperglycemia.”**

**Response: Done**

**- Line 494: Avoid redundancy. For instance, instead of stating “statins have the ability to,” simply state “statins can.”**

**Response: Done**

**2. Word Choices:**

**- The language is at times unnecessarily complex. Simplifying sentences can enhance clarity. For instance, instead of “in the context of vascular endothelial dysfunction-related disorders,” consider, “in conditions related to vascular dysfunction.”**

**Response: Done**

**- Avoid using overly broad terms like "marked positivity." Be specific about what is being measured and its significance rather than using vague descriptors.**

**Response: Done**

**- Lines 500-502: Terms such as "boosts antioxidant defenses" and "protect high density lipoprotein's antioxidant impact" should be defined more rigorously. It’s critical to clarify not just the effects, but the biological mechanisms at play.**

**Response: Done**

**As follows:**

“Previous studies demonstrated that statins have the ability to boost antioxidant defenses verified by the improvement of GSH and reduction of MDA)

Statins protected high density lipoprotein's antioxidant impact by increasing paraoxonase-1 (PON1) activity, which statin boosted in hypercholesterolemic patients”

**3. Redundancy:**

**- The phrase "which significantly influence AGE-dependent activation in DMC" could be streamlined to "which significantly influence AGE activation in DMC." Remove unnecessary qualifiers to enhance clarity and conciseness.**

**Response: Done**

**- Lines 499-500: The phrase "Interestingly, a former study documents..." is repetitive with "a previous study" used earlier. Choose one variation to maintain clarity.**

**Response: Done**

**4. Typographical Errors:**

**- Ensure there are no typographical errors such as “TL4” which should be corrected to “TLR4.” Also, “NOD-like protein 3;;” contains extraneous punctuation.**

**Response: Done**

**- The phrases “acute myocardial infarction” and “ischemia-reperfusion” require consistent hyphenation or formatting across the document.**

**Response: Done**

**- Lines 488-489: The transition between studies requires smoother transitions, rather than abrupt shifts. Each study reference should flow naturally into the next one. For instance, instead of starting a new sentence with “Moreover,” integrate it into the previous thought.**

**Response: Done**

**- Lines 492-493: Ensure consistent formatting for citations and ensure they are complete. For instance, “[60][61]” should be formatted in a standard referencing style used by the target journal.**

**5. Figures and References:**

**- Reference to figures must include clear associations between text and visual data. For instance, if Figure 14 is mentioned, a succinct interpretation of what the figure illustrates is required (“Figure 14 illustrates…”) to reinforce its importance.**

**Response: Done**

**- Lines 484-485: The text frequently references figure 14 without adequately describing what the figure represents. A brief explanation linking how the figure illustrates key points about the signaling pathways or protective mechanisms could enhance integration and understanding of the visual data presented.**

**Response: Done**

**- Ensure all citations are accurate and formatted consistently per journal guidelines.**

**Response: Done**

**VI. Discussion**

**1. Clarity and Structure:**

**- The discussion should be structured in a way that flows logically from one point to the next. This can be achieved by clearly separating different themes (e.g., mechanisms of action, implications for treatment, limitations, and future directions) into distinct paragraphs to enhance readability.**

**Response:** While restructuring the body of the discussion may not be feasible at this stage, we acknowledge the importance of clearly addressing limitations and future directions.

As follows:

**Limitations of the study:**

This study provides valuable insights into the protective effects of RVS and PTS in mitigating F/Fr/STZ-induced DCM, yet several limitations must be acknowledged. One significant limitation is the lack of data on the long-term effects of RVS and PTS in DCM, particularly concerning their safety and efficacy over extended periods. While the current findings highlight promising mechanisms, the translation of these results to clinical practice poses challenges, including differences between animal models and human physiology.

**Future Directions:**

Further research is needed to determine whether these effects can be replicated in clinical settings, especially among patients with varying stages of DCM or comorbidities such as hypertension and obesity. Investigating the potential interactions of RVS and PTS with other therapeutic agents and evaluating their impact on broader cardiovascular outcomes would also be essential for validating their therapeutic potential.

While this study provides compelling evidence for the potential of RSV and PTS in mitigating DCM in animal models, translating these findings to human patients requires careful consideration of several factors. In humans, dosing, treatment duration, and patient demographics (e.g., age, comorbidities such as diabetes or hypertension) will play crucial roles in determining the therapeutic efficacy and safety of RSV and PTS. Therefore, future research should focus on designing randomized controlled trials to assess the safety and efficacy of RSV and PTS in diverse human populations, taking into account variations in treatment responses. Moreover, studies should explore the long-term effects of RSV and PTS on cardiac function, fibrosis, and inflammation markers to establish their therapeutic potential in human DCM. Additionally, combining RSV and PTS with other therapeutic agents might yield synergistic effects, warranting further investigation into combination therapies.

**2. Reinforcement of Key Findings:**

**- The discussion should effectively reinforce the key findings presented in the results section. Each key point (e.g., the effects of RSV and PTS on cardiac function, oxidative stress, and inflammatory pathways) should directly correlate with the data presented earlier. Referring back to specific figures and tables to support claims would strengthen this section.**

**Response: Done**

**5. Mechanistic Insights:**

**- When discussing the impact on molecular signaling pathways (e.g., NLRP3 inflammasome, NF-κB/TLR-4), a more detailed explanation of the upstream triggers and downstream effects would be beneficial. For example, how do RSV and PTS influence reactive oxygen species production, leading to NLRP3 activation? Providing mechanistic insights can help readers understand the relevance of these pathways in DCM.**

**Response: Done**

**6. Statins in Clinical Context:**

**- The discussion should bridge the gap between the study findings and clinical applications. It should discuss the implications of using RSV and PTS in treating DCM in humans, considering factors such as dosing, patient demographics, and potential side effects. This would provide practical relevance to the research findings.**

**Response: Done**

***As follows in future research:***

While this study provides compelling evidence for the potential of RSV and PTS in mitigating DCM in animal models, translating these findings to human patients requires careful consideration of several factors. In humans, dosing, treatment duration, and patient demographics (e.g., age, comorbidities such as diabetes or hypertension) will play crucial roles in determining the therapeutic efficacy and safety of RSV and PTS. Therefore, future research should focus on designing randomized controlled trials to assess the safety and efficacy of RSV and PTS in diverse human populations, taking into account variations in treatment responses. Moreover, studies should explore the long-term effects of RSV and PTS on cardiac function, fibrosis, and inflammation markers to establish their therapeutic potential in human DCM. Additionally, combining RSV and PTS with other therapeutic agents might yield synergistic effects, warranting further investigation into combination therapies.

**7. Limitations:**

**- Addressing limitations is crucial for scientific integrity. The authors should discuss any limitations of their study, such as the animal model used (e.g., differences between rat and human physiology), duration of treatment, or sample size. Acknowledging these limitations can guide future research directions and help set realistic expectations for clinical translations.**

**Response:** Thank you for your insightful comment. The addressing the limitations of our study is important for scientific integrity and providing clear context for the findings. The following limitations have been added:

**Limitations of the study:**

This study provides valuable insights into the protective effects of RVS and PTS in mitigating F/Fr/STZ-induced DCM, yet several limitations must be acknowledged. One significant limitation is the lack of data on the long-term effects of RVS and PTS in DCM, particularly concerning their safety and efficacy over extended periods. While the current findings highlight promising mechanisms, the translation of these results to clinical practice poses challenges, including differences between animal models and human physiology.

**8. Future Research Directions:**

**- The discussion should end with a concise paragraph highlighting potential directions for future research. This could include suggestions for human clinical trials, exploring combinations with other therapeutic agents, or investigating long-term effects. Specifying these directions would demonstrate the relevance and potential impact of the study**

**Response:** Thank you for your insightful comment. The following future research has been added:

**Future Directions:**

Further research is needed to determine whether these effects can be replicated in clinical settings, especially among patients with varying stages of DCM or comorbidities such as hypertension and obesity. Investigating the potential interactions of RVS and PTS with other therapeutic agents and evaluating their impact on broader cardiovascular outcomes would also be essential for validating their therapeutic potential.

While this study provides compelling evidence for the potential of RSV and PTS in mitigating DCM in animal models, translating these findings to human patients requires careful consideration of several factors. In humans, dosing, treatment duration, and patient demographics (e.g., age, comorbidities such as diabetes or hypertension) will play crucial roles in determining the therapeutic efficacy and safety of RSV and PTS. Therefore, future research should focus on designing randomized controlled trials to assess the safety and efficacy of RSV and PTS in diverse human populations, taking into account variations in treatment responses. Moreover, studies should explore the long-term effects of RSV and PTS on cardiac function, fibrosis, and inflammation markers to establish their therapeutic potential in human DCM. Additionally, combining RSV and PTS with other therapeutic agents might yield synergistic effects, warranting further investigation into combination therapies.

**Reviewer #2:**

We greatly appreciate the reviewer’s thoughtful and constructive comments. Thank you for taking the time to review our manuscript; your suggestions have been incredibly helpful in enhancing both the clarity and quality of our work.

**The manuscript is well-organized and reads well, the study design is sound and appropriate for addressing the objectives**

**Major comments:**

**1- What is the rationale for studying the selected drugs (RVS, PTS), do they have certain advantage over the commonly prescribed statins such as (atorvastatin) and why atorvastatin was not included as a study group for comparison?**

**Response:** Thank you for your insightful question. The selection of Rosuvastatin (RVS) and Pitavastatin (PTS) for this study was based on several factors that distinguish these drugs from commonly prescribed statins like Atorvastatin. RVS and PTS were chosen due to their unique pharmacological profiles, which may offer distinct advantages in the context of diabetic cardiomyopathy (DCM).

Both RVS and PTS have demonstrated potent pleiotropic effects beyond lipid-lowering, particularly in terms of their anti-inflammatory, antioxidative, and cardioprotective properties. These effects may be particularly beneficial in conditions such as DCM, where inflammation, oxidative stress, and fibrosis play pivotal roles in disease progression. Additionally, RVS and PTS have shown greater potency in some studies compared to other statins in modulating signaling pathways associated with cardiac protection, such as the RISK pathway and NF-κB/NLRP3 inflammasome. Both RVS and PTS have been shown to exhibit strong lipid-lowering effects and may have unique anti-inflammatory properties compared to atorvastatin, making them particularly relevant for our research [22]. Additionally, previous studies have suggested that RVS and PTS may offer specific advantages in terms of tolerability, effects on endothelial function, and pleiotropic benefits beyond cholesterol lowering. For instance, RVS has been reported to have a more favorable effect on high-sensitivity C-reactive protein (hs-CRP) levels, an important marker for inflammation, while PTS has been noted for its potential superior effects in patients with diabetes or metabolic syndrome.

Given these differences, we felt it was more pertinent to focus on RVS and PTS, as they may provide insights into specific mechanisms or therapeutic advantages not fully addressed by atorvastatin. Nevertheless, we appreciate the reviewer’s suggestion, and we agree that a comparison with atorvastatin could provide additional context. We will consider including atorvastatin as a reference group in future studies to strengthen the comparison and broaden the clinical relevance of our findings.

The rational was added to the aim of the study as follows:

Rosuvastatin (RVS) and pitavastatin (PTS) are statin members that utilize several pharmacological activities and pleiotropic effects, including antioxidant, anti-inflammatory, and cardioprotective activities. Both RVS and PTS have shown potent effects in modulating various signaling pathways, including the RISK pathway, NF-κB/NLRP3 inflammasome, and TLR4/NF-κB signaling pathways. These pathways are critical in addressing the inflammation, oxidative stress, and fibrosis that contribute to disease progression in DCM [23]. Moreover, RVS and PTS have been shown to exhibit strong lipid-lowering effects and may have unique anti-inflammatory properties compared to atorvastatin, making them particularly relevant for our research (Morofuji and Nakagawa, 2022).

As for why Atorvastatin was not included, while it is a widely used and well-researched statin, our study aimed to focus on exploring the potential of RVS and PTS, which have not been as extensively studied in the context of DCM. The goal was to investigate whether these statins, with their distinct molecular actions, could offer additional therapeutic benefits. Including Atorvastatin in the comparison group is a valid consideration for future studies, but for the scope of this research, we focused on RVS and PTS due to their potential for offering new insights into treatment strategies for DCM.

Administration of ATOR for long time led to adverse degenerative effects on structure of the cardiac muscle fibres, although it has a potent lowering effect on lipid profile in hyperlipidemia [24].

**2- The authors claim that their study is the first to investigate the beneficial effects of RVS on DCM. However, a previous study by Luo et al. (PMID: 24254031), which shares similarities in study design, demonstrated the benefits of RVS in DCM through the NLRP3 inflammasome pathway. This prior work is not cited in the current study.**

**Response:** Thank you for pointing this out. We appreciate the reference to the study by Luo et al. (PMID: 24254031), which indeed shares similarities with our work. We acknowledge that this prior study also investigated the effects of RVS on DCM through the NLRP3 inflammasome pathway. We will include a proper citation to Luo et al. (2014) in the revised version of the manuscript and discuss the overlap with their findings as follows:

“Luo et al. (2014) attributed the positive impact of RSV on DCM to the inhibition of the NLRP3 inflammasome, which was associated with a reduction in MAPK activation [25].**”**

**Methods:**

**In general, the methodology is lacking sufficient description in some areas**

**- The strain of the rats used in the study has to be included**

**Response**: We were mentioned the strain of rats (adult male albino rats) in line 109 on page 4

**- A breakdown of the high fat/fructose diet composition is not provided**

**Response**: Done

**- Is a single reading taken one week after STZ administration sufficient to confirm induction of the diabetic state? Citing literature that supports the validity of verifying successful T2DM induction from a single reading is necessary.**

Thank you for your valuable comment. We understand the reviewer’s concern regarding the use of a single reading to confirm the successful induction of the diabetic state one week after STZ administration. While multiple readings are often used to confirm T2DM induction, a single reading can still be sufficient, provided it is carefully timed and follows established protocols.

Several studies have used a single glucose measurement within the first week post-STZ treatment to confirm diabetes induction. Our previous work demonstrated that a single glucose measurement after a standard induction period (7-10 days post-STZ) is typically sufficient to assess hyperglycemia and confirm diabetes induction in rodent models[10,17] . Additionally, Sharma et al. (2009) found that a single post-treatment glucose measurement could reliably differentiate between successfully induced diabetic animals and those that were not properly induced, particularly when using a threshold of 200-350 mg/dL or higher [26].

Previous work involved obtaining fasting blood samples after 2 days of STZ injection, and hyperglycemia was established to be greater than 300 mg/dL [27].

**- Was the manifestation of cardiomyopathy phenotype confirmed in the** **DCM group prior to treatment and how?**

**Response:** Thank you for your insightful question. Yes, the manifestation of the cardiomyopathy phenotype in the DCM group was confirmed prior to treatment. The presence of cardiomyopathy was assessed using a combination of diagnostic methods, including echocardiography to evaluate cardiac function and morphology, as well as histopathological analysis of heart tissue.

These assessments confirmed the presence of the cardiomyopathy phenotype prior to the administration of treatment, ensuring that the animals in the DCM group exhibited the expected pathophysiological features of the condition.

Moreover, this model of DCM is well established by previous studies. Several studies have used a single glucose measurement within the first week post-STZ treatment to confirm diabetes induction. Our previous work demonstrated that a single glucose measurement after a standard induction period (7-10 days post-STZ) is typically sufficient to assess hyperglycemia and confirm diabetes induction in rodent models [10,17].

**- The frequency of drug administration is not specified. It remains unclear whether the drugs were given once or twice daily.**

**Response:** Done, we were added that in abstract and in Study design on page 4 (DCM group treated orally with (RVS, 10 mg/kg) or for four weeks, and a DCM group treated orally with (PTS, 0.8 mg/kg) daily for four weeks).

**- The serum biochemical analysis is reported to have been conducted using a colorimetric assay; however, details on the kit, including its name and version, are missing. If these tests were performed by a service laboratory, this should be clearly stated.**

**Response:** Done

**- Details on the** **GSH/MDA assay kit, including its name and version, are missing. If the assays were conducted by a service laboratory, this should be clearly stated. Additionally,** **a full description of the protocol could be included in the supplementary material to facilitate reference and replication**

These details were mentioned in section of 2.6.3.1.	Cardiac Colorimetric Analysis in lines 168 and 169 on page 6 as follows:

Cardiac homogenates were used in the colorimetric estimation of reduced glutathione (GSH) (Biodiagnostic, GR 25 11, Egypt) and malondialdehyde (MDA) (Biodiagnostic, MD 25 29, Giza, Egypt) contents.

A full description of the protocol GSH/MDA assay kit, were added in the supplementary material

**- Line 171-172: “The reaction was concluded by heating at 99 °C for 5 min. Subsequently, the tubes containing the RT preparations…” The statement should be revised for scientific accuracy, the end product of reverse transcription process is cDNA not RNA**

**- Line 178: The statement “…to estimate the heart tissue samples of rat's copy number” is incorrect, as qPCR in this context measures gene expression, not gene copy number or gene dosage.**

**Response:** Done.

**- Line 180: primers direction is expressed as forward and reverse and not sense and antisense, the latter is used when working with RNA.**

**Response:** Done.

**- Line 182: Table 1 is missing, the equation for gene expression fold change using the ΔΔCt method should be formatted with the power function in superscript. Also, the name of the reference gene that was used for normalization is not indicated.**

**Response:** Done.

**- Line 185: “Aorta and heart samples were rapidly collected from rats in each group”, did the authors mean samples were collected rapidly after euthanasia?**

**Response:** Thank you for your question. Yes, when we stated that "aorta and heart samples were rapidly collected from rats in each group," we mean that the samples were collected immediately after euthanasia. The tissues were harvested quickly to preserve their integrity and prevent any changes that could affect the analysis.

**- At which step the MT staining was introduced in the tissue blocks preparation protocol mentioned elve**

**Response:** Thank you for your comment. The MT (Masson’s Trichrome) staining was introduced after the tissue blocks were sectioned and mounted on slides. Specifically, once the tissue blocks were formalin-fixed, paraffin-embedded, and sectioned, the slides were deparaffinized and rehydrated before the staining procedure. The staining was then performed according to standard protocols, allowing for the visualization of collagen deposition and other tissue features.

The detailed description is as follows:

Masson's trichrome stain is typically introduced during the process of tissue processing, after the tissue blocks have been properly fixed and embedded.

•	Thin sections of the embedded tissue are cut using a microtome.

•	Deparaffinization and Rehydration: The tissue sections are first deparaffinized using xylene, then rehydrated through a series of alcohol solutions, ending with water.

•	The rehydrated tissue sections are stained using the Masson’s trichrome staining protocol, which typically involves several stages. The process generally includes:

-Weigert's iron hematoxylin (for nuclear staining),

- Biebrich scarlet-acid fuchsin (for staining cytoplasm and muscle)

- Phosphotungstic acid-

- The sections are then washed and dehydrated again then mounted with a coverslip.

**- Line 213-215: information on the type/source of secondary Abs, incubation period, temperature (RT or 4C) and the manufacturer is missing**

**Response: Done**

**Results:**

**- What is the difference between DCM and DMC?**

**Response:** DCM (Diabetic Cardiomyopathy): refers to Diabetic Cardiomyopathy, a condition where diabetes leads to heart muscle dysfunction and remodeling, even in the absence of coronary artery disease or hypertension. It typically results in structural and functional changes in the heart, such as left ventricular dysfunction, fibrosis, and impaired contractility, often contributing to heart failure in diabetic patients.

On the other hand, DMC (Diabetes Mellitus Complications): refers to Diabetes Mellitus Complications, which encompass a range of health problems that arise due to uncontrolled or poorly managed diabetes. These complications can affect multiple organs, including the kidneys (diabetic nephropathy), eyes (diabetic retinopathy), nerves (diabetic neuropathy), and heart (leading to diabetic cardiomyopathy, among others).

**- Section 3.8: the results of the application of the scoring system (per Galati et al) are not described why?**

**Response: Done**

**- Figure 9: What is the anatomical location of the cardiac muscle? What is the number of animals from which tissue was analysed and number of slides analysed per animal and are they from the same block or different blocks?**

**Response:** In our study, we took sections from different cardiac sites either atrial or ventricle, left side or right side. The sections from three rats in each group mounted on 2 blocks and their prepared slides examined and analyzed.

**- Figure 11: scale bar indicating area of interest should be included in all images for clarity**

**Response: Done**

We adjusted all the scale bar of the figures.

**- Figures 12/13: The figure legend is missing explanations for what each panel represents.**

**Response:** Done

**Discussion**

**- The study does not address its limitations or drawbacks. Discussing these aspects would provide a more balanced view of the research findings**

**Response:** Thank you for your insightful comment. The addressing the limitations of our study is important for scientific integrity and providing clear context for the findings. The following limitations have been added.

**Limitations of the study:**

This study provides valuable insights into the protective effects of RVS and PTS in mitigating F/Fr/STZ-induced DCM, yet several limitations must be acknowledged. One significant limitation is the lack of data on the long-term effects of RVS and PTS in DCM, particularly concerning their safety and efficacy over extended periods. While the current findings highlight promising mechanisms, the translation of these results to clinical practice poses challenges, including differences between animal models and human physiology.

**- The difference in the histopathological effects of the two drugs is not discussed, nor are any speculations provided as to why this discrepancy occurs (Figures 9,10,12 C,D)**

**Response:** Thank you for your insightful comment. This part was added as follows:

“Furthermore, histopathological examination revealed normal myocardial architecture in the control group, with well-organized myofibrils and no signs of inflammation or fibrosis. In contrast, the F/Fr/STZ-induced DCM model showed significant inflammatory cell infiltration, collagen deposition, and fat accumulation in the myocardium, indicating fibrosis and structural damage. Treatment with RSV reduced inflammatory infiltration and partially restored myocardial structure, though some irregularities remained. PTS treatment resulted in more normalized myocardial architecture, with well-aligned myocardial fibers and a size closer to the control group, suggesting its effectiveness in mitigating cardiac damage. Both RSV and PTS demonstrated potential in improving myocardial structure and reducing inflammation in DCM. Together, these histopathological findings demonstrate the potential of both RSV and PTS in reversing the structural and inflammatory changes associated with DCM, with PTS showing a more normalized myocardial architecture compared to RSV. This fibrosis impairs heart function and contributes to heart failure. Treatment with RSV reduced fibrosis to a moderate degree, while PTS treatment resulted in minimal fibrosis and nearly normal myocardial architecture, with well-aligned fibers and restored cardiac myocyte size. These findings suggest that PTS is particularly effective in reducing fibrosis and preserving heart structure in DCM.

**- For completeness, the discussion could include a paragraph describing the effects of RVS and PTS on diabetic patients with cardiomyopathy secondary to T2DM or those with cardiometabolic diseases, as this would provide a broader perspective**

**Response: Done**

**6. PLOS authors have the option to publish the peer review history of their article (what does this mean?). If published, this will include your full peer review and any attached files.**

**If you choose “no”, your identity will remain anonymous but your review may still be made public.**

**Do you want your identity to be public for this peer review? For information about this choice, including consent withdrawal, please see our Privacy Policy.**

**Reviewer #1: No**

**Reviewer #2: No**

Journal requirements:

When submitting your revision, we need you to address these additional requirements.

1. Please ensure that your manuscript meets PLOS ONE's style requirements, including those for file naming. The PLOS ONE style templates can be found at

<https://journals.plos.org/plosone/s/file?id=wjVg/PLOSOne_formatting_sample_main_body.pdf> and

<https://journals.plos.org/plosone/s/file?id=ba62/PLOSOne_formatting_sample_title_authors_affiliations.pdf>

2. To comply with PLOS ONE submissions requirements, in your Methods section, please provide additional information regarding the experiments involving animals and ensure you have included details on (1) methods of sacrifice, (2) methods of anesthesia and/or analgesia, and (3) efforts to alleviate suffering.

3. Your ethics statement should only appear in the Methods section of your manuscript. If your ethics statement is writen in any section besides the Methods, please delete it from any other section.

**Response: Done**

4. Please include a copy of Tables 1 and 2 which you refer to in your text on pages 6 and 16.

**Response: Done**

**References**

[1]	J.J. Bax, G.B. Bleeker, T.H. Marwick, S.G. Molhoek, E. Boersma, P. Steendijk, E.E. van der Wall, M.J. Schalij, Left ventricular dyssynchrony predicts response and prognosis after cardiac resynchronization therapy, J Am Coll Cardiol 44 (2004) 1834–1840. https://doi.org/10.1016/j.jacc.2004.08.016.

[2]	K.E. Trinkley, R. Lee Page, H. Lien, K. Yamanouye, J.E. Tisdale, QT interval prolongation and the risk of torsades de pointes: essentials for clinicians, Curr Med Res Opin 29 (2013) 1719–1726. https://doi.org/10.1185/03007995.2013.840568.

[3]	M. Hanna, H. Seddiek, B.E. Aboulhoda, G.N.B. Morcos, A.M.A. Akabawy, M.A. Elbaset, A.A. Ibrahim, M.M. Khalifa, I.M. Khalifah, M.S. Fadel, T. Shoukry, Synergistic cardioprotective effects of melatonin and deferoxamine through the improvement of ferritinophagy in doxorubicin-induced acute cardiotoxicity, Front Physiol 13 (2022). https://doi.org/10.3389/fphys.2022.1050598.

[4]	S.A.E. Bashandy, A.M.A. El-Seidy, F.A.A. Ibrahim, S.S. Abdelrahman, S.A. Abdelmottaleb Moussa, M.A. ElBaset, Zinc nanoparticles ameliorated obesity-induced cardiovascular disease: role of metabolic syndrome and iron overload, Sci Rep 13 (2023) 16010. https://doi.org/10.1038/s41598-023-42550-y.

[5]	M.A. Elbaset, M. Nasr, B.M.M. Ibrahim, O.A.H. Ahmed‐Farid, R.M. Bakeer, N.S. Hassan, R.F. Ahmed, Curcumin nanoemulsion counteracts hepatic and cardiac complications associated with high‐fat/high‐fructose diet in rats, J Food Biochem 46 (2022) 1–16. https://doi.org/10.1111/jfbc.14442.

[6]	S.M. Al-Khatib, N.M.A. LaPointe, J.M. Kramer, R.M. Califf, What Clinicians Should Know About the QT Interval, JAMA 289 (2003). https://doi.org/10.1001/jama.289.16.2120.

[7]	L.U. Pagan, M.J. Gomes, M. Gatto, G.A.F. Mota, K. Okoshi, M.P. Okoshi, The Role of Oxidative Stress in the Aging Heart, Antioxidants 11 (2022) 336. https://doi.org/10.3390/antiox11020336.

[8]	M. Peng, Y. Fu, C. Wu, Y. Zhang, H. Ren, S. Zhou, Signaling Pathways Related to Oxidative Stress in Diabetic Cardiomyopathy, Front Endocrinol (Lausanne) 13 (2022). https://doi.org/10.3389/fendo.2022.907757.

[9]	N.G. Frangogiannis, Cardiac fibrosis, Cardiovasc Res 117 (2021) 1450–1488. https://doi.org/10.1093/cvr/cvaa324.

[10]	N.M.E. Abo El- Nasr, D.O. Saleh, I.M. Hashad, Role of olmesartan in ameliorating diabetic nephropathy in rats by targeting the AGE/PKC, TLR4/P38-MAPK and SIRT-1 autophagic signaling pathways, Eur J Pharmacol 928 (2022) 175117. https://doi.org/10.1016/j.ejphar.2022.175117.

[11]	E. Roudier, O. Mistafa, U. Stenius, Statins induce mammalian target of rapamycin (mTOR)-mediated inhibition of Akt signaling and sensitize p53-deficient cells to cytostatic drugs, Mol Cancer Ther 5 (2006) 2706–2715. https://doi.org/10.1158/1535-7163.MCT-06-0352.

[12]	J. Gollmer, A. Zirlik, H. Bugger, Established and Emerging Mechanisms of Diabetic Cardiomyopathy, J Lipid Atheroscler 8 (2019) 26. https://doi.org/10.12997/jla.2019.8.1.26.

[13]	H. V. Landa-Galvan, E. Rios-Castro, T. Romero-Garcia, A. Rueda, J.A. Olivares-Reyes, Metabolic syndrome diminishes insulin-induced Akt activation and causes a redistribution of Akt-interacting proteins in cardiomyocytes, PLoS One 15 (2020) e0228115. https://doi.org/10.1371/journal.pone.0228115.

[14]	X. Xu, Y. Hua, N. Sreejayan, Y. Zhang, J. Ren, Akt2 knockout preserves cardiac function in high-fat diet-induced obesity by rescuing cardiac autophagosome maturation, J Mol Cell Biol 5 (2013) 61–63. https://doi.org/10.1093/jmcb/mjs055.

[15]	C.-W. Liu, F. Yang, S.-Z. Cheng, Y. Liu, L.-H. Wan, H.-L. Cong, Rosuvastatin postconditioning protects isolated hearts against ischemia-reperfusion injury: The role of radical oxygen species, PI3K-Akt-GSK-3β pathway, and mitochondrial permeability transition pore, Cardiovasc Ther 35 (2017) 3–9. https://doi.org/10.1111/1755-5922.12225.

[16]	N. Kelley, D. Jeltema, Y. Duan, Y. He, The NLRP3 Inflammasome: An Overview of Mechanisms of Activation and Regulation, Int J Mol Sci 20 (2019) 3328. https://doi.org/10.3390/ijms20133328.

[17]	N.M.E. Abo El- Nasr, D.O. Saleh, S.S. Mahmoud, S.M. Nofal, R.M. Abdelsalam, M.M. Safar, H.S. El-Abhar, Olmesartan attenuates type 2 diabetes-associated liver injury: Cross-talk of AGE/RAGE/JNK, STAT3/SCOS3 and RAS signaling pathways, Eur J Pharmacol 874 (2020) 173010. https://doi.org/10.1016/j.ejphar.2020.173010.

[18]	M. Abdelbaset, M.M. Safar, S.S. Mahmoud, S.A. Negm, A.M. Agha, Red yeast rice and coenzyme Q10 as safe alternatives to surmount atorvastatin-induced myopathy in hyperlipidemic rats, Can J Physiol Pharmacol 92 (2014) 481–489. https://doi.org/10.1139/cjpp-2013-0430.

[19]	A. Sheridan, C.P.D. Wheeler-Jones, M.C. Gage, The Immunomodulatory Effects of Statins on Macrophages, Immuno 2 (2022) 317–343. https://doi.org/10.3390/immuno2020021.

[20]	B. Luo, B. Li, W. Wang, X. Liu, X. Liu, Y. Xia, C. Zhang, Y. Zhang, M. Zhang, F. An, Rosuvastatin alleviates diabetic cardiomyopathy by inhibiting NLRP3 inflammasome and MAPK pathways in a type 2 diabetes rat model., Cardiovasc Drugs Ther 28 (2014) 33–43. https://doi.org/10.1007/s10557-013-6498-1.

[21]	A.R. Bland, F.M. Payne, J.C. Ashton, T. Jamialahmadi, A. Sahebkar, The cardioprotective actions of statins in targeting mitochondrial dysfunction associated with myocardial ischaemia-reperfusion injury, Pharmacol Res 175 (2022) 105986. https://doi.org/10.1016/j.phrs.2021.105986.

[22]	Y. Morofuji, S. Nakagawa, K. Ujifuku, T. Fujimoto, K. Otsuka, M. Niwa, K. Tsutsumi, Beyond Lipid-Lowering: Effects of Statins on Cardiovascular and Cerebrovascular Diseases and Cancer, Pharmaceuticals 15 (2022) 151. https://doi.org/10.3390/ph15020151.

[23]	K. Koushki, S.K. Shahbaz, K. Mashayekhi, M. Sadeghi, Z.D. Zayeri, M.Y. Taba, M. Banach, K. Al-Rasadi, T.P. Johnston, A. Sahebkar, Anti-inflammatory Action of Statins in Cardiovascular Disease: the Role of Inflammasome and Toll-Like Receptor Pathways, Clin Rev Allergy Immunol 60 (2021) 175–199. https://doi.org/10.1007/s12016-020-08791-9.

[24]	N.M. Kadasa, H. Abdallah, M. Afifi, S. Gowayed, Hepatoprotective effects of curcumin against Diethyl nitrosamine induced hepatotoxicity in albino rats, Asian Pacific Journal of Cancer Prevention (2015). https://doi.org/10.7314/APJCP.2015.16.1.103.

[25]	Y. Xu, Y. Yang, Y.Q. Luo, Effect of atorvastatin on serum oxidative stress and N-terminal brain natriuretic peptide expression in rats, Asian Pac J Trop Med 7 (2014) 398–401. https://doi.org/10.1016/S1995-7645(14)60064-X.

[26]	A. Sharma, M. Vijayakumar, Ch. V. Rao, M.K. Unnikrishnan, G.D. Reddy, Action of Portulaca oleracea against Streptozotocin-Induced Oxidative Stress in Experimental Diabetic Rats, J Complement Integr Med 6 (2009). https://doi.org/10.2202/1553-3840.1181.

[27]	F.A. Hanan, A. Ebtissam, E. Mohamed, M. Hemaia, M. Faten, Attenuation of some Metabolic Deterioration Induced by Diabetes Mellitus using Nepeta cataria Extracts., Journal of American Science 6 (2010).
