# Supplementary material for: Role of rosuvastatin and pitavastatin in alleviating diabetic cardiomyopathy in rats: Targeting of RISK, NF-κB/ NLRP3 inflammasome and TLR4/ NF-κB signaling cascades
Source: PLoS One. 2025 Jul 30;20(7):e0325767. doi: 10.1371/journal.pone.0325767 (PMC12310044; doi:10.1371/journal.pone.0325767)
Supplement: Attachment — Submitted filename: Response to Reviewer Comments plos one cardiomyopathy.docx [file pone.0325767.s004.docx]

**Response to Reviewer Comments**

**Manuscript Title:** Role of Rosuvastatin and Pitavastatin in Alleviating Diabetic Cardiomyopathy in Rats: Targeting of RISK, NF-κB/ NLRP3 Inflammasome and TLR4/ NF-κB Signaling Cascades

**Manuscript Number:** PONE-D-24-48775R1

**Authors:** **Dalia O. Saleh, Nesma M.E. Abo El Nasr, Marawan A. Elbaset, Marwa E. Shabana, Tuba Esatbeyoglu, Sherif M. Afifi , Ingy M. Hashad**

***Reviewer #1: I have reviewed the revised manuscript PONE-D-24-48775_R1 and compared it to the initial round of comments. The authors have made significant improvements in addressing the concerns raised in the first review, including clarifying methodologies, refining statistical reporting, improving discussion points, and correcting language inconsistencies. However, some issues remain unresolved or only partially addressed, and these need further attention before the manuscript can be fully accepted.***

**Respond:** We sincerely appreciate the thoughtful and constructive feedback provided by the reviewers. Your insights have greatly enhanced our manuscript, and we are grateful for your time and consideration.

Reviewer's Responses to Questions

***Remaining Issues That Require Further Revision***

***1. Statistical Clarity and Effect Size Reporting (Lines 130–141)***

***-Unresolved Issue: While the authors have reported statistical significance (P-values), they have still not provided effect sizes (e.g., Cohen’s d, η², or odds ratios for logistic regression models), which are crucial for evaluating biological relevance.***

***-Suggested Fix:*** ***Include effect size measures alongside P-values to quantify the magnitude of observed differences.***

**Respond: The effect size was added within the supplementary data for each figure and expressed as** **r^2^ or eta-squared (η²). Also, we referred to the availability for this table in the statistical section in the manuscript.**

**As follows:**

| **Parameter** | **Effect size (eta-squared (η²).** |
| --- | --- |
| **Total Cholesterol** | 0.9174 |
| **Triglycerides** | 0.8620 |
| **Glucose** | 0.8719 |
| **RR Interval (s)** | 0.8886 |
| **Heart Rate (BPM)** | 0.9181 |
| **PR Interval (s)** | 0.4448 |
| **QRS Interval (s)** | 0.3874 |
| **QTc (s)** | 0.9548 |
| **R Amplitude (mV)** | 0.6970 |
| **ST Height (mV)** | 0.4609 |
| **NLRP3 (ng/mg protein)** | 0.9515 |
| **Cardiac MDA** | 0.9349 |
| **Cardiac GSH** | 0.7327 |
| **Cardiac pro-fibrotic IL-1 β** | 0.9410 |
| **cardiac Akt** | 0.8627 |
| **p-GSK-3β** | 0.8346 |
| **Cardiac NF-κB** | 0.9833 |
| **TLR-4** | 0.9390 |
| **cardiac troponin** | 0.9591 |

***2. Overinterpretation of Causal Relationships (Lines 142–200)
- Unresolved Issue: Despite some improvements, the discussion still implies causation in certain statements regarding statins' effects on NF-κB and NLRP3 inflammasome signaling.***

***-Suggested Fix: Replace phrases like "Statins reduce NLRP3 activation, leading to improved cardiac function" with "Statins are associated with reduced NLRP3 activation, which may contribute to improved cardiac function."***

**Respond**: **Done**

***3. Insufficient Acknowledgment of Confounding Factors (Lines 201–216)***

***- Unresolved Issue: The authors have not sufficiently discussed how patient heterogeneity, pre-existing inflammation, or medication history might influence their findings.***

***-Suggested Fix: Add a brief acknowledgment of potential confounders and recommend future studies to control for these variables.***

**Respond**: We appreciate the reviewer’s concern regarding the acknowledgment of confounding factors such as patient heterogeneity, pre-existing inflammation, and medication history. However, we would like to clarify that our study was conducted as a preclinical experiment using animal models (rats) and not human patients. Consequently, the factors of patient heterogeneity and medication history are not directly applicable in our study.

In our preclinical model, we controlled confounding variables through inclusion/exclusion criteria for the animals according to serum glucose levels in the induction of diabetic model. Additionally, pre-existing inflammation was considered by the inclusion of normal rats in the beginning of the experiment and control groups in the experimental design. While these factors may be relevant in clinical settings, they do not directly influence the interpretation of our findings within the context of our preclinical design. We hope this clarification addresses the reviewer’s concerns. Thank you for your thoughtful feedback***.***

***4. Lack of Clarity on Missing Data Handling (Lines 100–129)***

***- Unresolved Issue: The manuscript still does not specify how missing values were handled in statistical analyses. Were missing values imputed or excluded?***

***- Suggested Fix: Clearly state the approach used (e.g., multiple imputation, listwise deletion, or mean substitution).***

**Respond**: **This sentence was added to the manuscript: “****If there are any missing data before analysis, we handled using listwise deletion, wherein cases with missing values were excluded from the respective analyses.”**

***5. Ambiguous Language and Grammar Issues***

***- Many language issues have been addressed, but some awkward phrasings remain.***

***- Example: Line 31: "The Dried Blood Spot (DBS) technique using filter papers is an easy, quick, transport-ease technique..."***

***- Fix: "The DBS technique is a rapid and convenient micro-sampling method using filter papers."***

**Respond**: **We revised the whole manuscript for any ambiguous terms.**

***Reviewer #2: A. The authors have adequately addressed almost all raised points, only a few were partially resolved:***

***1. the frequency of the daily drug dose is still not explicitly stated (is the drug administered once or twice daily)***

**Respond**: **We appreciate the reviewer’s observation regarding the frequency of drug administration. To clarify, the drug was administered once daily for four consecutive weeks.**

We have revised the manuscript to explicitly state this information in the relevant section In abstract: the RVS group of DCM-induced rats that were treated once daily with 10mg/kg of RVS, and the PTS group of DCM rats that were treated with 0.8 mg/kg of PTS).

In materials and methods section:

2.4.	Study design:

Rats confirmed to have diabetes were randomly assigned into three groups: an untreated diabetic cardiomyopathy (DCM) group, a DCM group treated orally with (RVS, 10 mg/kg) or (PTS, 0.8 mg/kg) once daily for four weeks. In addition to another group being fed a standard diet with free access to water which served as a normal control. At week seven, these rats received a citrate buffer injection (1 ml/kg, i.p.)

Thank you for pointing this out, and we hope this clears up any confusion.

***2. the full definition of the term DMC should be introduced early on in the manuscript***

**Respond**: **The abstract and introduction sections now begin with this term (**Diabetic cardiomyopathy (DCM))

***B. Some of the responses will enhance the clarity of the manuscript if incorporated where appropriate into the main text or added to the supplementary material:***

***1. The response to "Is a single reading taken one week after STZ administration sufficient...."***

**Respond**: We would like to clarify that blood glucose levels were measured at two time points:

The first measurement was taken one week after STZ administration and served only as an inclusion criterion to confirm the successful induction of diabetes and to select diabetic rats for the study.

The second measurement was taken at the end of the experiment to evaluate the persistent hyperglycemia in the diabetic group and assess the effect of the treatments.

We have added to the manuscript’s main text (Methods section, under Experimental Design/Animal Grouping):

"Blood glucose levels were measured at two time points: initially, one week after STZ administration, to confirm diabetes induction for group allocation; and again at the end of the experimental period to assess the maintenance of hyperglycemia and treatment effects."

***2. MT staining steps can be added to the supplementary material***

**Respond**: We have added as Supplementary File 1:

MT staining steps can be added to the supplementary material.

We have prepared a detailed, step-by-step Masson's Trichrome staining protocol to be included in the Supplementary Material for full transparency and reproducibility.

***3. Response to comments on Fig9 can be incorporated into the figure legend***

**Respond**: We revised the legend of Figure 9 to include an explanatory note based on the reviewer’s comments, providing clarity on how the measurements were obtained and interpreted.

We added the following part to the legend of Figure 9:

*"*Figure 9 illustrates the histological examination of myocardial tissue, including evaluation of inflammatory cell infiltration, fibrosis, and fat accumulation characteristic of DCM. The observed changes were semi-quantitatively assessed and supported by Masson’s Trichrome staining for collagen deposition. Treatment with RSV showed partial improvement, while PTS treatment more effectively preserved myocardial architecture, reduced fibrosis, and restored myocyte size closer to the control group values*."*
